# Problematic Facebook use and problematic video gaming as mediators of relationship between impulsivity and life satisfaction among female and male gamers

**DOI:** 10.1371/journal.pone.0237610

**Published:** 2020-08-18

**Authors:** Andrzej Cudo, Marcin Wojtasiński, Przemysław Tużnik, Mark D. Griffiths, Emilia Zabielska-Mendyk

**Affiliations:** 1 Department of Experimental Psychology, The John Paul II Catholic University of Lublin, Lublin, Poland; 2 International Gaming Research Unit, The Nottingham Trent University, Nottingham, United Kingdom; University of Auckland, NEW ZEALAND

## Abstract

Over the past few decades, many new technologies have emerged, such as portable computers, the internet and smartphones, which have contributed to improving the lives of individuals. While the benefits of these new technologies are overwhelmingly positive, negative consequences are experienced by a minority of individuals. One possible negative aspect of new technologies is their problematic use due to impulsive use which may lead to lower life satisfaction. The present study investigated the mediating role of problematic video gaming (PVG) and problematic Facebook use (PFU) in the relationship between impulsivity dimensions and life satisfaction as well as the relationship between impulsivity dimensions and problematic behaviors. Additionally, the potential impact of gender differences was also examined. The study comprised 673 gamers (391 females) aged 17–38 years (M = 21.25 years, SD = 2.67) selected from 1365 individuals who completed an offline survey. PFU was assessed using the Facebook Intrusion Scale, and PVG was assessed using the nine-item Internet Gaming Disorder Scale–Short-Form (IGDS9-SF). Impulsivity dimensions such as attention, cognitive instability, motor, perseverance, self-control, and cognitive complexity were assessed using the Barratt Impulsiveness Scale (BIS-11), and life satisfaction was assessed using the Satisfaction With Life Scale (SWLS). Depending on the specific impulsivity dimension, findings showed both positive and negative relationships between impulsivity and life satisfaction. Attention and perseverance subtypes of impulsivity were primarily associated with problematic behaviors. Additionally, cognitive complexity was associated with PFU among female gamers, whereas cognitive instability was associated with PVG among male gamers. Additionally, PVG was primarily associated with lower life satisfaction. However, there was no mediation effects between impulsivity dimensions and life satisfaction via PFU or PVG. These findings provide a better understanding of the relationship between problematic behaviors, life satisfaction, and impulsivity among gamers and the differences between male and female gamers.

## Introduction

Over the past few decades, many new technologies have emerged including portable computers, the internet and smartphones. Furthermore, there is little doubt that such technologies have improved the lives of individuals all around the world [1, 2]. Internet and smartphones provide the capabilities to for individuals to contact almost anyone anywhere in the world. In this context, social media has become a new platform for interpersonal relationships [3]. Additionally, computers and smartphones from devices primarily designed for work purposes have also become a tool for entertainment (such as online gaming), and the number of online gamers has increased year-on-year [4]. However, despite the many positive developments related to new technologies, they can also have potential negative effects among a small minority, such as problematic social media (i.e., Facebook) use and problematic video gaming (PVG). Consequently, many scholars are interested in understanding the mechanisms underlying these behaviors, as well as the relationship between these problematic behaviors and life satisfaction, and between personal individual differences such as impulsivity and these problematic behaviors. In this context, an important issue is the mutual relationship between problematic behaviors associated with videogame playing and Facebook use, impulsivity, and life satisfaction.

Videogames have been defined as “a mode of interaction between a player, a machine with an electronic visual display, and possibly other players, that is mediated by a meaningful fictional context, and sustained by an emotional attachment between the player and the outcomes of her actions within this fictional context” [5, p. 25], are very popular worldwide. According to the forecast adjusted for the expected impact of COVID-19 on the worldwide videogame market segment [6], there were 1.46 billion videogame users in 2019. The Statista Global Consumer Survey released in October 2019 indicates that individuals aged 25 to 34 years comprised the largest group of videogame players (36.4%) and 61% of videogame players were males [6]. Additionally, the popularity of videogames is also very high in the Polish population. According to the most recent report, in 2019, 76% of internet users in Poland were videogame players (20.9 million) [7]. According to the report of the Polish Gamers Observatory, in 2019, individuals aged 25 to 34 years comprised the largest group of players (32%) and 53% of Polish gamers were males [8].

According to report on worldwide Facebook use, in June 2020, Facebook was the most popular social networking platform compared to Instagram, Pinterest, YouTube, Tumblr, Reddit, and Twitter [9]. In the first quarter of 2020, there were 2.6 billion active monthly users of Facebook [10]. In April 2020, 56% of Facebook users were male [11] and individuals aged 25 to 34 years were the largest segment in the user group (32%) [12]. Statistics concerning Facebook use among the Polish population are similar to those worldwide ones. The report of Polish Facebook use indicated that in June 2020, Facebook was the most popular social networking platform in Poland compared to Instagram, Pinterest, YouTube, Tumblr, Reddit and Twitter [13]. Almost 51.4% of the Polish population used Facebook, and individuals aged 25 to 34 years comprised the largest group (26.7%) [14]. The same report noted that 53.5% of Facebook users were female. Consequently, playing videogames and Facebook use are among the most frequent activities engaged in on the internet.

Taking into account the popularity of Facebook and videogames, individuals spend a lot of time using these media. Sometimes, the frequent use of such media may change into problematic use [15, 16]. Additionally, problematic Facebook use (PFU) and PVG have been correlated with low life satisfaction [17–19]. Particularly interesting, in the context of PVG and PFU, is the issue of personality traits (i.e., impulsivity) and their impact upon life satisfaction. Consequently, it can be assumed that PFU and PVG may have potential mediating role in relationship between impulsivity and life satisfaction. Additionally, taking into account difference between male and female Facebook users and videogame players [20], gender may differentiate the aforementioned relationships.

## Impulsivity

Impulsivity has been investigated from various perspectives, and several definitions have been proposed. Eysenck [21] characterized impulsivity as unplanned risky behaviors and making up one’s mind quickly. Impulsivity may also be defined “as a predisposition toward rapid, unplanned reactions to internal or external stimuli without regard to the negative consequences of these reactions to the impulsive individual or to others” [22, p. 1784]. Additionally, DeYoung and Rueter [23, p. 348] characterized impulsivity trait as “a tendency to act on immediate urges, either before consideration of possible negative consequences or despite consideration of likely negative consequences”. In this context, DeYoung and Rueter [23] also point out that impulsive action consists of at least two elements: (i) an impulse–an urge, motivation, or desire–to act in some way, and (ii) a lack of inhibition, restraint, or control of that impulse.

However, it should also be noted that impulsivity is not a one-dimensional construct [24, 25]. Patton, Stanford, and Barratt [24] classify impulsivity trait into three dimensions: (i) non-planning impulsivity which is a tendency to plan and think without deliberation, (ii) attentional impulsivity which is difficulties in focusing on a task and cognitive activities, and (iii) the motor impulsivity which is a tendency to act on the spur of the moment. Moreover, other previous research [25, 26] indicated that impulsivity might also have the following dimensions: (i) negative urgency which is the tendency to act rashly under extreme negative emotions, (ii) positive urgency which is the tendency to act rashly under extreme positive emotions, (iii) lack of premeditation which is the tendency to act without thinking, (iv) lack of perseverance which is the inability to remain focused on a task, and (v) sensation seeking which is the tendency to seek out novel and thrilling experiences. Consequently, impulsivity should be considered as a multidimensional construct, which can be related to addictive behaviors in various ways.

## Impulsivity and life satisfaction

Taking into account that impulsivity is a predisposition toward rapid, unplanned reactions without regard to the negative consequences [22], impulsivity can be expected to have a direct or indirect relationship with life satisfaction. In this context, Figueira et al. [27], as well as Porto et al. [28], reported a negative association between general impulsivity and life satisfaction. Additionally, Goodwin et al. [29], as well as McKewen et al. [30], showed that general impulsivity was negatively associated with wellbeing. Poorer quality of life was also significantly explained by higher attentional, motor, and non-planning impulsivity [31]. Similarly, Caron et al. [32] reported a negative association between general impulsivity and quality of life. However, previous research reported no statistically significant relationship between general impulsivity and life satisfaction [33, 34], and between life satisfaction and impulsive sensation seeking [35].

Furthermore, Arrindell, Heesink and Feij [36] reported a weak positive correlation between impulsivity characterized as habitual response style of decision making in ambiguous situations and life satisfaction among male and female groups. However, their results also showed a negative association between life satisfaction and disinhibition characterized by the desire to find release through social disinhibition, drinking alcohol, and going to parties, irrespective of gender. Taken together, it can be assumed that impulsivity may contribute to low satisfaction, but this relationship has not been clarified. Consequently, it is essential to verify the direct and indirect relationship between impulsivity and life satisfaction, considering behaviors such as PFU and PVG.

## Problematic Facebook use and problematic video gaming

PFU may be defined as an excessive involvement in Facebook, disrupting day-to-day activities and interpersonal relationships [37]. Additionally, PFU is characterized by losing control over Facebook use and developing a strong psychological need to stay online, despite the possible negative consequences of this behavior [38]. It should also be noted that PFU is a specific example of problematic social media use because Facebook users can watch videos, play games, gamble online, share photos, update their profiles, stream live videos, and message their friends [39, 40]. Consequently, Facebook offers more possibilities than most other social media applications such as WhatsApp, Instagram, and Twitter.

PVG has become a topic of increasing interest among public health professionals. Gaming disorder was included in the latest (eleventh) revision of the International Classification of Diseases (ICD-11) and defines the behavior as being “characterized by a pattern of persistent or recurrent gaming behavior (…) manifested by: (1) impaired control over gaming (…); (2) increasing priority given to gaming to the extent that gaming takes precedence over other life interests and daily activities; and (3) continuation or escalation of gaming despite the occurrence of negative consequences” [41]. Additionally, problematic gaming was included in the latest (fifth) edition of the Diagnostic and Statistical Manual of Mental Disorders (DSM-5) in Section III (Condition for Further Study) as ‘Internet Gaming Disorder’ (IGD) [42]. The proposed symptoms of IGD include: (i) preoccupation with internet games, (ii) withdrawal symptoms when internet gaming is taken away, (iii) tolerance, (iv) unsuccessful attempts to control the participation in internet games, (v) loss of interests in previous hobbies and entertainment as a result of, and with the exception of, internet games, (vi) continued excessive use of internet games despite knowledge of psychosocial problems, (vii) has deceived family members, therapists, or others regarding the amount of internet gaming, (viii) use of internet games to escape or relieve a negative mood (e.g., feelings or helplessness, guilt, anxiety), and (ix) has jeopardized or lost a significant relationship, job, or educational or career opportunity because of participation in internet games. Scholars such as Griffiths [43–45] stipulate that six specific symptoms (i.e., salience, mood modification, tolerance, withdrawal, conflict and relapse) must be present in order for any behavior such as gaming and social media use to be considered an addiction. However, problematic behavior may still be present even if some of these symptoms and consequences are not present. In the present study, the PVG and PFU are considered in line with the theoretical framework proposed by Griffiths [43–45].

According to the Interaction of Person-Affect-Cognition-Execution (I-PACE) model [15, 16], PFU, and PVG may be considered as a subtype of addictive behavior (see [16]). According to this model, behavioral specific predisposing variables such as specific needs, specific motives, and specific values, in conjunction with the general predisposing variables such as genetics, early childhood experiences, psychopathology, coping strategies, and temperamental features, may condition a specific type of problematic behavior. In this context, Brand et al. [15] pointed out that impulsivity trait may be one of the important predictors of problematic behavior. Additionally, according to the I-PACE model, the conjunction of behaviorally specific predisposing variables and general predisposing variables influence external and internal triggers associated with specific problematic behaviors.

These triggers may also be related to experiencing gratification at the beginning of media use. Consequently, users experiencing gratification may spend increasing amounts of time on using media such as videogames and Facebook. However, this gratification decreases as addiction progresses. Additionally, as addiction progresses, in place of the gratification, compensation for the negative effects of the problematic behaviors arises. In this situation, the users can also spend increasing amounts of time on using the media because they need to compensate for the negative consequences associated with problematic media use. Brand et al. [16] also noted that general inhibition control might moderate the relationship between affective and cognitive responses to these triggers. In this context, previous research [46] has reported the relationship between inhibition control and impulsivity. Additionally, Wegmann et al. [47] reported that increased symptom severity of problematic social media use was associated with higher attentional impulsivity, especially when there were additional reductions in executive functions or specific inhibitory control. However, it should be noted that previous studies have indicated that playing videogames may lead to different cognitive functioning [48, 49]. Consequently, gamers (compared to non-gamers) may have a different cognitive capacity, which may theoretically modify the cognitive reactivity to triggers associated with specific subtypes of addictive behavior.

Taking into account the I-PACE model [15, 16], it can be assumed that impulsivity is one of the crucial predictors of problematic behaviors. Additionally, many studies point to high impulsivity as one of the essential risk factors of psychoactive substance use [50–52] and behavioral addictions [53–58]. Previous studies have also indicated that high impulsivity is associated with low life satisfaction [27] and that addictions are also associated with low life satisfaction [59–61]. Also, there appears to be differences between males and females in relation to addictive behaviors [20, 62] as well as impulsivity [63]. Consequently, it is essential to understand better the relationship between problematic behaviors, life satisfaction, and impulsivity among male and female gamers. More specifically, on one hand, it is important to examine the relationship between multidimensional impulsivity traits and problematic behaviors. On the other hand, it is essential to verify that problematic behaviors such as PFU and PVG are mediators in the relationship between impulsivity and life satisfaction. Moreover, given the shortage of research on the specificities of female gamers' functioning, it is essential to clarify these relationships in this gamer group.

## Impulsivity and PFU and PVG

In a PFU context, Cudo et al. [64] examined the relationship between action control, impulsivity, restraint, and PFU among 234 individuals (91.5% female; M = 24.86 years). They reported that PFU was positively associated with impulsivity characterized by a tendency to act spontaneously without deliberation [65]. Additionally, there was a positive relationship between performance-related action orientation and PFU, whereas no statistically significant relationship between restraint and PFU. Similarly, previous studies [66, 67] based on the reflective–impulsive theory of the mind [68], showed that PFU is primarily associated with dysfunction of the impulsive system which is responsible for generating impulsive behavior, rather than the reflective system which serves regulatory goals and is responsible for higher-order mental operations.

Khoury et al. [69] reported a positive relationship between PFU and general impulsivity. Moreover, Rothen et al. [70] reported a positive relationship between positive urgency, negative urgency, lack of perseverance, and PFU whereas there was no statistically significant relationship between lack of premeditation, sensation seeking, and PFU. Additionally, Orosz et al. [71] reported that PFU was associated with high urgency and lack of perseverance. However, they did not examine other impulsivity dimensions. Walker et al. [72] reported no statistically significant relationship between negative urgency and Facebook intensity characterized as an emotional connection to Facebook and the incorporation of Facebook into individuals’ daily life. However, they did not verify other impulsivity dimensions. Taken together, previous research [64, 66, 67, 69] indicates a relationship between impulsivity and PFU. However, the results of other studies [70–72] suggest that not all dimensions of impulsivity are associated with PFU.

Previous studies have shown that impulsivity is associated with PVG [58, 73]. In this context, Blinka, Škařupová and Mitterova [74] reported a positive relationship between PVG, game engagement, frequency of play, and dysfunctional impulsivity. Nuyens et al. [75] experimentally investigated the relationship between impulsivity and PVG considering the different dimensions of impulsivity (self-description and experimental). They reported that there was no statistically significant relationship between positive urgency, negative urgency, lack of premeditation, lack of perseverance, sensation seeking, motor impulsivity, non-planning impulsivity, and PVG. However, PVG was positively associated with attentional impulsivity and negatively with the mean waiting time during the first half of the single key impulsivity experimental task.

Additionally, Rømer Thomsen et al. [76] reported no statistically significant relationship between PVG and every impulsivity dimension such as urgency, lack of premeditation, lack of perseverance, and sensation seeking. Similarly, Deleuze et al. [77] showed no difference between PVG group and health control group (in this same impulsivity dimensions examined by Rømer Thomsen et al. [76]. Bargeron and Hormes [17] reported a higher level of attentional and motor impulsivity among gamers with PVG compared to gamers without PVG. However, they no found the difference between groups in non-planning impulsivity.

Choi et al. [78] showed that individuals with PVG had higher attentional impulsivity than the control group. In contrast, they presented lower attentional impulsivity then individuals with problematic gambling. Additionally, the PVG group displayed higher motor impulsivity than the control group and problematic gambling group. Patients with PVG displayed higher non-planning impulsivity than the problematic gambling group. Sariyska et al. [79] reported no statistically significant relationship between PVG and impulsivity traits such as attentional, motor, and non-planning impulsivity among female gamers, whereas there was only a positive correlation between motor impulsivity and PVG among male gamers. Additionally, they reported no statistically significant relationship between PVG and all impulsivity traits among male World of Warcraft (WoW) players, whereas there was only a negative correlation between motor impulsivity and PVG among female WoW players.

Taken together, it should be noted that the relationship between one-dimensional impulsivity and PVG is well recognized [58]. However, previous research [17, 75–78] demonstrates that when different dimensions of impulsivity are taken into account, the results of relationship analysis between PVG and impulsivity dimensions is not consistent. In this context, one possible explanation for the different research results may be the small sample sizes and gender bias. Consequently, it is essential for future research to investigate the relationship between impulsivity dimensions and problematic behaviors by including larger sample sizes and greater gender representation. Additionally, it should not be forgotten that impulsivity and addictive behaviors are also associated with life satisfaction which is characterized as an individual’s judgement of the quality of their life on the basis on their own unique criteria [80, 81]. Consequently, understanding the relationship between impulsivity dimensions and problematic behaviors such as PFU and PVG may lead to a more detailed clarification of the specific relationship between impulsivity and life satisfaction mediated by PFU and PVG.

## Life satisfaction and PFU and PVG

Previous research [17–19] has reported negative relationships between problematic behavior and life satisfaction. More specifically, Marino et al. [19] examined the associations between PFU, psychological distress (i.e., depression, anxiety, etc.) and wellbeing (life satisfaction, positive mental health) among adolescents and young adults in a meta-analysis of 23 studies. They reported that PFU was negatively associated with life satisfaction, and (in general) positively associated depression and anxiety. Additionally, the results of the meta-regression showed that the relationship between PFU and psychological distress was larger in older samples. Similar to Marino et al. [19], a meta-analysis of 23 studies showed that problematic internet use had a negative effect on the quality of life [18].

In PVG, Bargeron and Hormes [17] reported that individuals with PVG have lower life satisfaction compared to gamers without PVG. Additionally, Montag et al. [82] reported a negative association between life satisfaction and problematic internet use among first-person-shooter video gamers. Similarly, Mentzoni et al. [83] reported that gamers with PVG had a lower level of life satisfaction, higher anxiety and higher depression compared to gamers without PVG and non-gamers. Mettler, Mills and Heath [84] and Lemmens, Valkenburg, and Peter [85] also reported a negative relationship between PVG and life satisfaction. Additionally, Pontes [86] reported that problematic social networking site (SNS) use and PVG are positively correlated to one another. The study also showed that both problematic behaviors might simultaneously influence overall psychological health such as depression, anxiety and stress. However, Tian et al. [87] reported that PVG correlated negatively with life satisfaction, whereas problematic social media use did not correlate with life satisfaction.

Taken together, and based on present research, it can be assumed that behaviors such as PFU and PVG may be negatively associated with life satisfaction. In this context, it should also be noted that Yu and Shek [61] tested longitudinal relationships between problematic Internet use and life satisfaction. Their findings supported the hypothesis that poor life satisfaction among adolescents is the consequence rather than the cause of problematic internet use. Taken together, and based on the studies presented, it can be assumed that problematic behaviors may be negatively associated with life satisfaction. Consequently, it is essential to examine the relationship between these variables with regard to impulsivity. More specifically, taking into account the relationship between impulsivity and life satisfaction [27–35], it can be assumed that problematic behaviors such as PFU and PVG may mediate the relationship between impulsivity and life satisfaction. However, taking into account the differences between males and females in terms of problematic online behaviors [20, 62], possible gender differences should be taken into account.

## Gender perspective

Previous research has indicated that the differences between males and females not only concern the prevalence of problematic online behaviors but also variables that may be associated with these problematic behaviors. Lachmann et al. [88] reported that despite higher problematic internet use levels among males than females, a negative association between life satisfaction and online problematic behavior was significantly higher among females than males. Similarly, Lei, Chiu and Li [89] reported that females had a stronger negative association between life satisfaction, subjective positive emotion, and problematic internet use in a meta-analysis of 70 Chinese studies.

Additionally, Martínez-Loredo et al. [90] identified latent profiles of individuals using different substances and gambling activities, including gender and impulsivity. They reported that general impulsivity was an essential predictor of class membership such as cigarette smokers with alcohol abuse and exclusively among male gamblers. Among females, general impulsivity was an essential predictor of class membership, such as cigarette smokers with alcohol abuse and broad users. Additionally, non-planning impulsivity was a predictor of exclusive gamblers among males.

However, Li et al. [91] reported that general impulsivity was positive associated with problematic internet use, irrespective of gender. Similarly, Shokri, Potenza and Sanaeepour [92] postulated similar relationships between problematic Internet use and impulsivity traits such as negative urgency, positive urgency, lack of perseverance, sensation seeking lack of premeditation across gender. However, Chen, Lo and Lin [93] reported a positive relationship between motor impulsivity and problematic internet use among females, whereas no similar relationship was found among males. In this context, it should be noted that Cross, Copping and Campbell [94] reported that males had a higher level of attentional, motor, non-planning, and perseverance impulsivity than females in meta-analysis of 56 studies. Taken together, it can be assumed that gender can be a moderator of the relationship between impulsivity and problematic behavior as well as and life satisfaction and problematic behaviors such as PVG and PFU. However, previous studies [88–95] have reported a non-consistent pattern of gender differences in these relationships. Consequently, it is important to understand the differences between males and females in the relationship between impulsivity and problematic behavior as well as life satisfaction and these types of behaviors.

## The present study

Previous studies have demonstrated the importance of the association between high impulsivity trait and behaviors such as PFU and PVG. However, to date, the relationships between these two problematic behaviors and the different impulsivity trait dimensions have not been thoroughly investigated. Taking into account previous studies [70, 71] it can be assumed that PFU is probably associated with lack of perseverance which is characterized as an inability to remain focused on a task (see [25, 26]). Similarly, Wegmann et al. [47] showed that increased symptom severity of problematic social media use was associated with higher attentional impulsivity which is described as difficulties in focusing on a task and cognitive activities (see [24]). Additionally, previous research [17, 75, 78] showed the relationship between attentional impulsivity and PVG. Consequently, it can be assumed that the difficulty of focusing on the task may be significant for both problematic behaviors.

In a motor impulsivity context, some studies have reported an association between this impulsivity dimension and problematic online behavior [17, 78]. However, other research has shown that this relationship was moderated by gender [79] or no statistically significant relationship between these variables [75]. Consequently, it can be very cautiously assumed that the motor impulsivity may be associated with PVG. Additionally, taking into account previous research [93] showing only a positive relationship between motor impulsivity and problematic internet use for females and the relationship between PVG and social media use [95], it can be assumed very cautiously that motor impulsivity will also be associated with PFU. Previous research [70, 71] found no association between PFU and lack of premeditation, characterized as a tendency to act without thinking (see [25, 26]). Similarly, Rømer Thomsen et al. [76] and Deleuze et al. [77] reported no statistically significant relationship between PVG and lack of premeditation.

Additionally, a previous study [17, 79] reported no statistically significant relationship between PVG and non-planning impulsivity characterized as a tendency to plan and think without deliberation (see [24]). Taking into account that lack of premeditation associated with non-planning impulsivity [25], it can be assumed that this dimension of impulsivity is not associated with PFU or PVG. Moreover, previous studies showed the relationship between problematic online behaviors and life satisfaction [17, 19, 83] as well as between impulsivity and life satisfaction [27, 28]. However, the results concerning the association between impulsivity and life satisfaction are inconclusive [27, 28, 33, 34]. Notably, the relationship between impulsivity dimensions and satisfaction is not clear [31, 35, 36]. In this context, and taking into account previous research reporting the relationship between impulsivity and problematic behaviors such as PVG and PFU [58, 64, 66, 67, 69] and the relationship between these behaviors and low life satisfaction [17–19], it can be assumed that PVG and PFU mediate the relationship between impulsivity and life satisfaction. Considering the gender differences in the relationship between problematic behaviors and impulsivity dimensions [79, 93] as well as between problematic behaviors and life satisfaction [88, 90], it should also be considered whether the pattern of these relationships is similar for both genders. Consequently, taking into account the theoretical considerations and aforementioned findings, the following hypotheses were formulated:

H1: Higher attentional impulsivity will be associated with both PFU and PVG.
H2: There will be a positive relationship between motor impulsivity and both PFU and PVG.
H3: There will be no relationship between PFU and PVG and the non-planning impulsivity.
H4: Higher problematic behaviors will be associated with lower life satisfaction.
H5: Impulsivity will be associated with life satisfaction.
H6: PVG and PFU will mediate the relationship between impulsivity and life satisfaction.
H7: The relationship between both PFU and PVG and impulsivity dimensions will be different among males compared to females.

Additionally, based on I-PACE model [15, 16], PFU and PVG may be treated as subtypes of specific internet-use disorder. Consequently, it can be assumed that, despite the differences related to the subject matter of the problematic behavior, PVG and PFU may have similar mechanisms related to the formation of the problematic behavior. Moreover, the I-PACE model [15, 16] also postulates that amount of time spent using videogames and Facebook may be linked to gratification or compensation mechanisms. Consequently, some studies have postulated that the amount of time spent on Facebook is a predictor of PFU [96, 97], while other studies have suggested that the time spent using Facebook is a consequence of PFU [98, 99]. An analogous situation can be noted in studies examining the relationship between gaming time and PVG [100–103]. Therefore, taking into account the I-PACE model [15, 16], findings from previous research [96–103], and the different models of the relationship between PVG and PFU, that the amount of time spent using videogames and Facebook should be verified to select the model that best describes the relationship between these variables. This exploratory research is important because previous studies [96–103] have mostly focused on the presentation of single models, without considering possible alternative models in line with I-PACE model.

## Materials and methods

### Participants

The study comprised 673 gamers (391 female gamers) aged 17–38 years (M = 21.25 years, SD = 2.67) selected from 1365 individuals who completed an offline survey. The criterion for inclusion of selected participants was based on whether they had played videogames (online and offline) in the past year. The female gamers’ ages ranged from 17 to 38 years (M = 21.04; SD = 2.59) and male gamers’ ages ranged from 17 to 35 years (M = 21.54; SD = 2.82)(t_(671)_ = -2.42; *p* = 0.016; Cohen`s *d* = 0.18). All participants were volunteers, and received no monetary reward for participation. They were from six voivodships (administrative regions) of Poland (Małopolskie, Mazowieckie, Kujawsko-Pomorskie, Lubelskie, Lubuskie and Wielkopolskie). The study was conducted in accordance with the Declaration of Helsinki. Participants were informed that their responses would be anonymous and confidential, and oral informed consent was obtained. The study was approved by the Institute of Psychology’s ethics committee (John Paul II Catholic University of Lublin) (Additionally, it should be noted that the present study was a part of a larger research project on psychological aspects of problematic behaviors such as PFU and PVG among young Polish adults. The research project has two main, broad parts. The first one relates to the analysis of the relationship between multidimensional aspects of impulsivity and problematic behaviors, and the second one relates to the analysis of the relationship between early maladaptive schemas and this type of behaviors. Consequently, taking into account the breadth and consistency of the issues, only the variables concerning the relationship between multidimensional aspects of impulsivity and problematic behaviors among Polish young adult gamers are examined in the present study. Additionally, the issue of the relationship between the early maladaptive schemas and problematic behaviors was reported in another manuscript, which has been submitted to another journal.).

### Measures

#### Problematic videogame playing

The nine-item Internet Gaming Disorder Scale–Short-Form (IGDS9-SF) [104] in a Polish version [105] was used to assess PVG. Items (e.g., “*Do you feel more irritability*, *anxiety or even sadness when you try to either reduce or stop your gaming activity*?”) are responded to on a five-point scale, from 1 (*strongly disagree*) to 5 (*strongly agree*). Higher scores reflect a greater intensity of PVG. The scale has good psychometric properties, with Cronbach’s alpha of 0.85 in the present study.

#### Problematic Facebook use

The eight-item Facebook Intrusion Scale [37] in a Polish version [106] was used to asses PFU. Items (e.g., “*I often think about Facebook when I am not using it*”) are responded to on a seven-point scale, from 1 (*strongly disagree*), to 7 (*strongly agree*). Higher scores reflect a greater intensity of PFU. The scale has good psychometric properties, with a Cronbach’s alpha of 0.84 in the present study. The scale has also been used in studies designed to investigate PFU in Poland [20, 107] and different countries such as China, Greece, Israel, Italy, Poland, Romania, Turkey and the USA [106].

#### Impulsivity

The Barratt Impulsivity Scale (BIS 11) [24, 63] in a Polish version [108] was used to assess impulsivity. It has three second-order factors (i.e., non-planning impulsivity, attentional impulsivity, and motor impulsivity). However, there are six first-order factors (i.e., attention, cognitive instability, motor, perseverance, self-control and cognitive complexity). Attention (five items; e.g., “*I don’t pay attention*”) and cognitive instability (three items; e.g., “*I often have extraneous thoughts when thinking*”) are a part of attentional impulsivity whereas motor (seven items; e.g., “*I act “on impulse*”) and perseverance (four items; e.g., “*I can only think about one thing at a time*”) are a part of motor impulsivity. Additionally, self-control (six items; e.g., “*I say things without thinking*”) and cognitive complexity (five items; e.g., “*I am more interested in the present than the future*”) are a part of non-planning impulsivity. Items are scored on a four-point scale from 1 (*rarely/never*) to 4 (*almost always/always*). Higher scores reflect higher intensity of impulsivity. In the original version, the subscale Cronbach alphas were 0.72 for attention, 0.55 for cognitive instability, 0.64 for motor, 0.27 for perseverance, 0.72 for self-control, and 0.48 for cognitive complexity [63]. In the present study, the subscale Cronbach alphas were 0.49 for attention, 0.45 for cognitive instability, 0.72 for motor, 0.33 for perseverance, 0.65 for self-control, and 0.41 for cognitive complexity.

#### Life satisfaction

The five-item Satisfaction With Life Scale [33] in a Polish adaptation [109] was used to assess life satisfaction. Items (e.g., “*In most ways*, *my life is close to my ideal*”) are responded to on a seven-point Likert scale, from 1 (*strongly disagree*) to 5 (*strongly agree*). Higher scores reflect greater life satisfaction. In the present study, the Cronbach’s alpha was 0.85.

#### Demographic variables and frequency of use

Participants also provided information concerning socio-demographic factors (age, gender), the time spent playing videogames per week and the time spent using Facebook per week.

### Statistical analysis

The descriptive statistics such as means and standard deviations for all the variables as well as Spearman correlation coefficients between the variables were calculated. In order to assess the differences between male and female gamers and taking into account the non-normal distribution of variables, the Mann-Whitney two-sample test [110] were applied [see S1 Fig]. Additionally, descriptive statistics such as means (M), standard deviations (SD), median (Me) and quartile deviation (Q) were presented for both groups. The magnitude of differences was assessed by η^2^ effect size [111].

In order to verify the relationship between impulsivity dimensions, PFU, PVG and life satisfaction, as well as the mediation effect between impulsivity and life satisfaction via PFU and PVG among male and female gamers, structural equation modelling was used. Taking into account previous studies [17, 64], a model with impulsivity dimensions as predictors of PFU and PVG was developed. Additionally, considering previous studies [28], the model included the relationship between impulsivity and life satisfaction. PFU and PVG were considered as predictors of life satisfaction in the model (see [17, 112]). Additionally, the relationship between Facebook hours and PFU as well as game hours and PVG was included (see [20]).

However, taking into account the I-PACE model [15, 16] and the differences in the models including relationships between problematic behaviors (i.e., PVG and PFU), and amount of time spent on Facebook or videogames [96–103], eight different models were examined [see S1 Table]. More specifically, the models with and without the relationship between PFU and PVG as well as with and without the relationship between time spent on Facebook and gaming were tested. Additionally, the models treating the time spent on Facebook and gaming as predictors or consequences of problematic behavior were compared. Additionally, the model with and without relationship between time spent on Facebook and gaming was tested. Taking into account all the aforementioned conditions presented, eight different models were tested [see S1 Table].

Structural equation model was based on the maximum likelihood method with Sattora-Bentler adjustment [113]. This adjustment was used because there was a violation of multivariate normal distribution between the female (Doornik–Hansen omnibus test (χ^2^_(df = 26) =_ 14802.03; *p*<0.001), Henze–Zirkler’s consistent test (χ^2^_(df = 1) =_ 4046.65; *p*<0.001), Mardia’s multivariate kurtosis test (χ^2^_(df = 1) =_ 1587.66; p<0.001) and Mardia’s multivariate skewness test (χ^2^_(df = 220) =_ 5122.37; *p*<0.001)) and male sample (Doornik–Hansen omnibus test (χ^2^_(df = 26) =_ 7759.28; *p*<0.001), Henze–Zirkler’s consistent test (χ^2^_(df = 1) =_ 2595.36; *p*<0.001), Mardia’s multivariate kurtosis test (χ^2^_(df = 1) =_ 346.85; *p*<0.001) and Mardia’s multivariate skewness test (χ^2^_(df = 220) =_ 2372.52; *p*<0.001)).

The following measures were used to assess the goodness of the model: χ^2^, χ^2^/df, RMSEA (Root Mean Square Error of Approximation), SRMR (Standardized Root Mean Squared Residual), CFI (Confirmatory Fit Index) and TLI (Tucker-Lewis Index). Statistically non-significant (*p*>0.05) chi-square values may suggest that the proposed model fits the dataset well. The value of chi-square/df ratio is lower than two and suggests a good fit to the dataset. Likewise, values of RMSEA and SRMR lower than 0.05 show a good fit of the model. Values of CFI and TLI higher than 0.95 indicate the model fits the dataset well [114, 115]. Additionally, the following metrics: the Akaike information criterion (AIC) [116], Schwarz's Bayesian information criterion (BIC) [117] and adjusted Schwarz's Bayesian information criterion (SSBIC) [118] were used to select the most informative model. Lowest information criterion values indicate the most informative model. Based on the analysis, the best model was selected and presented.

In order to test differences between male and female gamers in regression weights, the Wald test [119] was conducted [120]. The mediation effects between impulsivity, time spent on Facebook and gaming, and problematic behaviors were assessed by the Zhao, Lynch, Chen [121] approach with Monte Carlo method (5000 samples) to estimate standardized indirect effects with 95% confidence interval [122]. The mediation effect was interpreted in accordance with Zhao, Lynch, Chen [121, p. 200] guidelines: (i) *complementary mediation*: indirect effect and direct effect both exist and point at the same direction; (ii) *competitive mediation*: indirect effect and direct effect both exist and point in opposite directions; (iii) *indirect-only mediation*: indirect effect exists, but no direct effect (full mediation); (iv) *direct-only non-mediation*: direct effect exists, but no indirect effect; and (v) *no-effect non-mediation*: neither direct effect nor indirect effect exists. The Statistical calculations were conducted using the statistical software IBM SPSS 23 for description statistic and correlation analysis, and Stata 14 with medsem ado package [122] for structural equation analysis and mediation analysis.

## Results

The descriptive statistics such as means and standard deviations, and correlation coefficients are presented in Table 1. The results showed that PFU was positively correlated with time spent on Facebook, PVG, and the impulsivity dimensions of attention, cognitive instability, motor, self-control, cognitive complexity, and total. Additionally, there was a positive relationship between time spent game and PVG. Also, PVG positively correlated with impulsivity dimensions of attention, cognitive instability, motor, perseverance, self-control, and total. There was a negative relationship between PVG and life satisfaction. Detailed findings are presented in Table 1.

**Table 1:** Mean values, standard deviations and Spearman’s rho correlation coefficients between analyzed variables.

Variables		M	SD	[1]	[2]	[3]	[4]	[5]	[6]	[7]	[8]	[9]	[10]	[11]
[1] Problematic Facebook use		20.24	8.75											
[2] Facebook number of hours per week		21.51	26.50	0.47***										
[3] Problematic video gaming		15.36	5.94	0.18***	-0.01									
[4] Game number of hours per week		12.04	16.72	0.01	0.13**	0.58***								
Impulsivity	[5] Attentional—Attention	10.43	2.52	0.20***	0.06	0.15***	0.03							
	[6] Attentional—Cognitive instability	7.50	1.81	0.15***	0.04	0.10*	-0.03	0.30***						
	[7] Motor—Motor	15.14	3.87	0.15***	0.05	0.15***	0.08*	0.32***	0.39***					
	[8] Motor—Perseverance	7.46	2.00	0.06	0.05	0.18***	0.14***	0.30***	0.17***	0.27***				
	[9] Nonplanning—Self control	12.85	3.06	0.14***	0.06	0.10**	0.06	0.41***	0.19***	0.51***	0.22***			
	[10] Nonplanning—Cognitive complexity	11.19	2.54	0.17***	0.08*	0.02	-0.02	0.37***	0.11**	0.29***	0.32***	0.36***		
	[11] Total	10.43	2.52	0.23***	0.09*	0.16***	0.05	0.67***	0.50***	0.77***	0.50***	0.74***	0.61***	
[12] Life satisfaction		21.46	6.05	0.01	0.08*	-0.14***	-0.09*	-0.16***	-0.22***	-0.03	-0.17***	-0.11**	-0.12**	-0.16***

*** *p* < .001

** *p* < .01

* *p* < .05

Further analysis showed the differences between female gamers and male gamers in PVG. More specifically, PVG was higher among males than females. Additionally, there was a difference in weekly time spent gaming between both groups. Male gamers spent more hours gaming than female gamers. The effect size of these differences was medium. Moreover, the findings showed the differences between male and female gamers on the impulsivity dimensions of attention and perseverance. Male gamers had higher impulsivity associated with these dimensions than female gamers. However, the effect size of these differences was small. Detailed findings are presented in Table 2.

**Table 2:** Differences between male (n = 282) and female (n = 391) gamers.

Variables		Male gamers (N = 282)				Female gamers (N = 391)				U	z	p	η^2^
		M	SD	Me	Q	M	SD	Me	Q				
Problematic Facebook use		19.88	9.02	18.00	5.50	20.50	8.55	19.00	5.50	51784.50	-1.35	0.178	0.003
Facebook number of hours per week		18.27	21.80	10.00	7.50	23.84	29.23	12.00	11.00	51448.00	-1.48	0.138	0.003
Problematic video gaming		17.94	6.07	17.00	4.50	13.49	5.10	12.00	3.00	29489.00	-10.35	0.001	0.159
Game number of hours per week		18.82	18.72	14.00	10.00	7.15	13.12	3.00	3.00	27396.00	-11.17	0.001	0.185
Impulsivity	Attentional–Attention	10.70	2.60	10.00	2.00	10.24	2.44	10.00	2.00	49987.00	-2.08	0.037	0.006
	Attentional–Cognitive instability	7.40	1.97	7.00	1.50	7.57	1.69	8.00	1.50	51655.50	-1.42	0.157	0.003
	Motor–Motor	15.39	3.75	15.00	2.50	14.96	3.95	15.00	3.00	51180.50	-1.59	0.111	0.004
	Motor–Perseverance	7.87	2.16	8.00	1.50	7.17	1.81	7.00	1.00	44579.00	-4.30	0.001	0.027
	Nonplanning–Self control	13.11	2.84	13.00	2.00	12.66	3.20	13.00	2.00	50305.50	-1.95	0.051	0.006
	Nonplanning–Cognitive complexity	11.20	2.53	11.00	2.00	11.19	2.54	11.00	2.00	54673.50	-0.19	0.853	0.001
	Total	65.66	11.28	64.00	8.00	63.80	10.23	63.00	7.00	50544.50	-1.84	0.065	0.005
Life satisfaction		21.30	5.93	22.00	3.63	21.57	6.15	22.00	4.50	53982.50	-0.46	0.644	0.001

M = arithmetic mean; SD = standard deviation; Me = median; Q = quartile deviation

**Fig 1:**
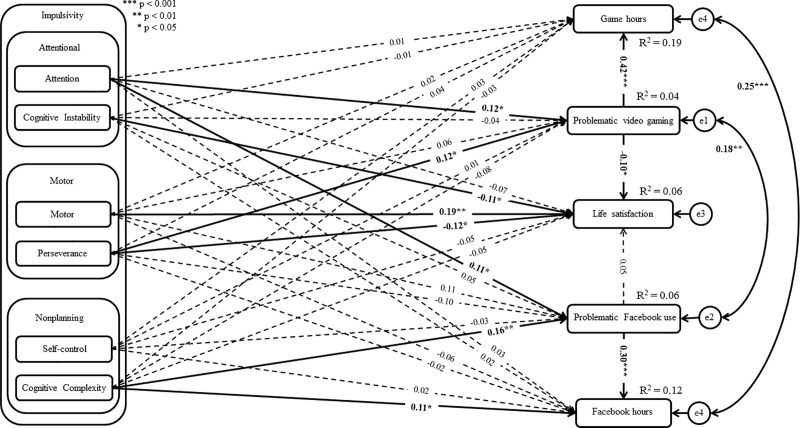
Structural model of the relationship between variables in female gamers group.

**Fig 2:**
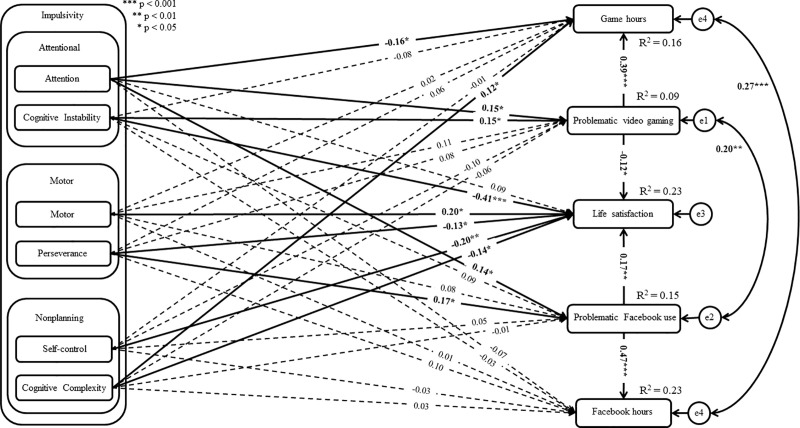
Structural model of the relationship between variables in male gamers group.

The analysis showed that two models out of eight had a good fit to the data (see Table 3: Model 2 and Model 6). Both models assumed a correlation between residuals of both problematic online behaviors as well as between the residuals of time spent on Facebook and gaming. However, in Model 2, time spent on Facebook and gaming was treated as a predictor of PFU and PVG, while in Model 6 these hours were included as consequences of PFU and PVG. Consequently, the AIC, BIC, and SSBIC information criterion was used to compare both models. The values of AIC, BIC, and SSBIC were lower for Model 6 than for Model 2. Given that models with lower AIC, BIC, and SSBIC are considered to be more informative, the results can be interpreted as showing that Model 6 was better than Model 2. Additionally, taking into account guidelines by Burnham and Anderson [123], the difference between AIC scores higher than 4 may suggest that Model 2 had considerably less support then Model 6. Consequently, Model 6 assuming time spent on Facebook as a consequence of problematic behavior as well as time spent gaming as a consequence of PVG was further considered (see Figs 1 and 2).

**Table 3:** Fit indices for eight models.

Model number	Condition I	Condition II	Condition III	Fit indices										
				χ^2^	Df	p<	χ^2^/df	CFI	RMSEA	SRMR	TLI	AIC	BIC	SSBIC
1	Facebook hours and game hours as a predictor of problematic behavior	Facebook and game hours residuals correlated	PFU and PVG residuals no correlated	37.89	10	0.001	3.79	0.929	0.091	0.021	0.434	43092.06	43741.75	43284.54
2			PFU and PVG residuals correlated	11.73	8	0.164	1.47	0.991	0.037	0.014	0.905	43063.45	43722.16	43258.60
3		Facebook and game hours residuals no correlated	PFU and PVG residuals no correlated	56.87	12	0.001	4.74	0.886	0.105	0.034	0.240	43121.59	43762.25	43311.39
4			PFU and PVG residuals correlated	33.85	10	0.001	3.39	0.939	0.084	0.028	0.516	43092.97	43742.66	43285.45
5	Facebook hours and game hours as a results of problematic behavior	Facebook and game hours residuals correlated	PFU and PVG residuals no correlated	26.87	10	0.003	2.69	0.957	0.071	0.024	0.657	43079.76	43729.45	43272.24
**6**			**PFU and PVG residuals correlated**	**7.87**	**8**	**0.446**	**0.98**	**1.000**	**0.001**	**0.012**	**1.003**	**43059.37**	**43718.09**	**43254.52**
7		Facebook and game hours residuals no correlated	PFU and PVG residuals no correlated	56.87	12	0.001	4.74	0.886	0.105	0.034	0.240	43121.59	43762.25	43311.39
8			PFU and PVG residuals correlated	39.97	10	0.001	4.00	0.924	0.094	0.024	0.391	43101.20	43750.89	43293.68

PFU = problematic Facebook use; PVG = problematic video gaming

The selected model highlighted in bold typeface.

Among female gamers, PFU was associated with the impulsivity dimensions of attention (β = 0.11; *p* = 0.039) and cognitive complexity (β = 0.16; *p* = 0.002), while PVG was associated with the impulsivity dimensions of attention (β = 0.12; *p* = 0.028) and perseverance (β = 0.11; *p* = 0.023). Additionally, there was a negative relationship between life satisfaction and the impulsivity dimensions of cognitive instability (β = -0.11; *p* = 0.042) and perseverance (β = -0.12; *p* = 0.035). Also, life satisfaction was negatively related with PVG (β = -0.10; *p* = 0.040) and positively related with motor impulsivity (β = 0.19; *p* = 0.002). There was a positive relationship between PFU, cognitive complexity (β = 0.11; *p* = 0.045), and the time spent using Facebook per week (β = 0.30; *p*<0.001). Additionally, the time spent gaming per week was associated with PVG (β = 0.42; *p*<0.001). The results also showed a significant relationship between the time spent on Facebook and gaming residuals (r = 0.25; *p*<0.001), as well as between PFU and PVG residuals (r = 0.18; *p* = 0.002). Detailed findings are presented in Fig 1 and the S2 Table.

For male gamers, PFU was associated with the impulsivity dimensions of attention (β = 0.14; *p* = 0.040) and perseverance (β = 0.17; *p* = 0.014), whereas PVG was associated with the impulsivity dimensions of attention (β = 0.15; *p* = 0.032) and cognitive instability (β = 0.15; *p* = 0.018). Additionally, PFU was associated with the time spent using Facebook per week (β = 0.47; *p*<0.001). There was a significant and positive relationship between PVG (β = 0.39; *p*<0.001), cognitive complexity (β = 0.12; *p* = 0.036), and the time spent gaming per week. Additionally, the impulsivity dimension of attention was negatively associated with time spent gaming (β = -0.16; *p* = 0.024). Moreover, there was a negative relationship between life satisfaction and the impulsivity dimensions of cognitive instability (β = -0.41; *p*<0.001), perseverance (β = -0.13; *p* = 0.035), self-control (β = -0.20; *p* = 0.005), and cognitive complexity (β = -0.14; *p* = 0.048). PVG was negatively associated with life satisfaction (β = -0.12; *p* = 0.033), while PFU was positively associated with life satisfaction (β = 0.17; *p* = 0.009). Additionally, there was a positive association between life satisfaction and motor impulsivity (β = 0.20; *p* = 0.012). Among female gamers, results showed that relationship between time spent on Facebook and gaming residuals (r = 0.27; *p*<0.001) as well as between both FPU and PVG residuals (r = 0.20; *p* = 0.003) were significant. Detailed findings are presented in Fig 2 and the S2 Table.

The difference analysis (using Wald’s test) showed regression weight differences between male and female gamers in the relationship between PFU and perseverance (χ^2^_(df = 1)_ = 9.43; *p* = 0.002). The standardized regression weight was non-significant among female gamers (β = -0.10, *p* = 0.066), while this relationship was significant among male gamers (β = 0.17, *p* = 0.014). In the case of the relationship between PFU and cognitive complexity, significant differences between the groups were found (χ^2^_(df = 1)_ = 4.17; *p* = 0.041). The standardized regression weight was significant among female gamers (β = 0.16; *p* = 0.002) whereas this path was significant among male gamers (β = -0.01, *p* = 0.832). There was a difference between the groups in the relationship between PFU and the time spent using Facebook per week (χ^2^_(df = 1)_ = 4.95; *p* = 0.026). The standardized regression weight was higher among male gamers (β = 0.47, *p*<0.001) than among female gamers (β = 0.30, *p*<0.001). Additionally, in the case of the relationship between PVG and cognitive instability, significant differences between the groups were found (χ^2^_(df = 1)_ = 4.25; *p* = 0.039). The standardized regression weight was significant among male gamers (β = 0.15, *p* = 0.018) and non-significant among female gamers (β = -0.04, *p* = 0.562). There was a difference between the groups in the relationship between the time spent gaming per week and impulsivity associated with attention (χ^2^_(df = 1)_ = 4.03; *p* = 0.045). The standardized regression weight was significant among male gamers (β = -0.16; *p* = 0.024) and non-significant among female gamers (β = 0.01, *p* = 0.839). In the case of the relationship between the time spent gaming per week and cognitive complexity, significant differences between the groups were found (χ^2^_(df = 1)_ = 3.96; *p* = 0.047). This path was significant among male gamers (β = 0.12; *p* = 0.036) and non-significant among female gamers (β = -0.03, *p* = 0.557). Additionally, there was a difference between the groups in the relationship between life satisfaction and cognitive instability (χ^2^_(df = 1)_ = 13.16; *p*<0.001). The standardized regression weight was significantly higher among male gamers (β = -0.41, *p*<0.001) than among female gamers (β = -0.11, *p* = 0.042). The other difference between male and female gamers in terms of analyzed paths was statistically non-significant (see Figs 1 and 2).

Based on the mediation analyses, the significant mediation effects of PFU and PVG on time spent on Facebook and gaming were found. Among female gamers, the findings showed a statistically significant indirect effect between cognitive complexity and time spent on Facebook PFU. Additionally, there was a direct significant effect between this impulsivity dimension and time spent on Facebook. Consequently, these results may indicate complementary mediation (partial mediation) (see [121]) among female gamers. There was a statistically significant indirect effect between perseverance and the time spent gaming per week via PVG, which may indicate full mediation. Additionally, the findings showed a statistically significant indirect effect between impulsivity associated with attention and time spent gaming via PVG in both groups. However, there was a direct significant effect between this impulsivity dimension and time spent gaming among male gamers, whereas this direct effect was non-significant among female gamers. Consequently, these results may indicate competitive mediation (partial mediation) (see [121] form more details) among male gamers and indirect-only mediation (full mediation) (see [121]) among female gamers. Among male gamers, there was a statistically significant indirect effect between cognitive instability on time spent gaming via PVG, which may indicate full mediation. Detailed findings are presented in Table 4.

**Table 4:** Standardized indirect effects with 95% confidence intervals.

Female gamer group						
Model pathways	Point estimates	Standard error	95%CI		*z*	*p*
			Lower	Upper		
Attention–PFU–Facebook hours	0.033	0.017	0.002	0.068	1.94	0.053
Perseverance–PFU–Facebook hour	-0.031	0.018	-0.067	0.001	-1.77	0.077
**Cognitive complexity–PFU–Facebook hours**	**0.046**	**0.017**	**0.015**	**0.082**	**2.74**	**0.006**
**Attention–PVG–game hours**	**0.049**	**0.023**	**0.005**	**0.095**	**2.12**	**0.034**
Cognitive instability–PVG–game hours	-0.017	0.029	-0.073	0.038	-0.59	0.553
**Perseverance–PVG–game hours**	**0.051**	**0.023**	**0.007**	**0.097**	**2.19**	**0.029**
Attention–PFU–life satisfaction	0.006	0.007	-0.005	0.022	0.86	0.391
Perseverance–PFU–life satisfaction	-0.006	0.007	-0.021	0.005	-0.85	0.398
Cognitive complexity–PFU–life satisfaction	0.008	0.009	-0.007	0.027	0.96	0.340
Attention–PVG–life satisfaction	-0.012	0.008	-0.031	0.001	-1.41	0.159
Cognitive instability–PVG–life satisfaction	0.004	0.008	-0.010	0.022	0.52	0.601
Perseverance–PVG–life satisfaction	-0.012	0.009	-0.032	0.001	-1.43	0.152
Male gamer group						
Attention–PFU–Facebook hours	0.065	0.034	0.003	0.134	1.95	0.052
**Perseverance–PFU–Facebook hour**	**0.081**	**0.035**	**0.016**	**0.153**	**2.13**	**0.021**
Cognitive complexity–PFU–Facebook hours	-0.007	0.031	-0.068	0.054	-0.23	0.821
**Attention–PVG–game hours**	**0.057**	**0.028**	**0.005**	**0.116**	**2.01**	**0.044**
**Cognitive instability–PVG–game hours**	**0.060**	**0.037**	**0.010**	**0.117**	**2.20**	**0.028**
Perseverance–PVG–game hours	0.032	0.027	-0.019	0.086	1.17	0.243
Attention–PFU–life satisfaction	0.023	0.015	-0.001	0.058	1.52	0.129
Perseverance–PFU–life satisfaction	0.028	0.017	0.002	0.066	1.70	0.090
Cognitive complexity–PFU–life satisfaction	-0.003	0.012	-0.028	0.021	-0.21	0.832
Attention–PVG–life satisfaction	-0.018	0.012	-0.046	0.001	-1.42	0.155
Cognitive instability–PVG–life satisfaction	-0.019	0.012	-0.047	0.001	-1.50	0.134
Perseverance–PVG–life satisfaction	-0.010	0.010	-0.033	0.006	-0.97	0.334

PFU = problematic Facebook use; PVG = problematic video gaming

## Discussion

The present study investigated the relationship between PFU, PVG, life satisfaction, and impulsivity dimensions among male and female gamers. Additionally, the mediation effect between impulsivity and life satisfaction was also tested. The findings showed that higher attention impulsivity (a subtype of attentional impulsivity) was associated with higher PFU and PVG, irrespective of gender. Additionally, higher cognitive instability was only associated with PVG among male gamers. There was a positive relationship between perseverance (motor impulsivity subtype) and (i) PVG among female gamers, and (ii) PFU among male gamers. However, there was no statistically significant relationship between the motor (a subtype of motor impulsivity) and both PFU and PVG, irrespective of gender.

Additionally, the results of the present study showed that higher cognitive complexity (non-planning impulsivity subtype) was associated with PFU only among female gamers. Apart from this relationship, there was no other statistically significant relationship between the subtypes of non-planning impulsivity and either PFU and PVG among male and female gamers. Additionally, the findings showed a negative relationship between PVG and life satisfaction in both groups. However, PFU was positively associated with life satisfaction among male gamers. There was a relationship between several impulsivity dimensions and life satisfaction. Also, the findings showed the difference between both groups in the relationship between perseverance, cognitive complexity, and the PFU as well as between PVG and cognitive instability. However, despite the significant relationship between impulsivity and problematic behaviors, and between life satisfaction and these behaviors, the results of the present study showed no mediation effect between impulsivity and life satisfaction via PVG or PFU.

As hypothesized, higher attentional impulsivity was associated with PFU and PVG (H1). Notably, the relationships between both problematic online behaviors and subtypes of attentional impulsivity (such as attention impulsivity) were significantly positive among male and female gamers. However, the relationship between the subtype of attentional impulsivity, such as cognitive instability and problematic online behaviors was only significant in the case of PVG among male gamers. This finding may indicate that difficulties in focusing on a task [24, 63] may contribute to increased problems with Facebook use and gaming, irrespective of gender.

These results were in line with previous research [70, 71] where PFU was associated with lack of perseverance, characterized as the inability to remain focused on a task [25, 26]. Also, Wegmann et al. [47] showed that increased symptom severity of problematic social media use was associated with higher attentional impulsivity. Additionally, previous research [17, 75, 78] reported a positive relationship between attentional impulsivity and PVG. According to the I-PACE model [16], attentional impulsivity may moderate the relationship between affective and cognitive responses to external or internal triggers associated with a specific behavior and decisions to involve in this behavior. More specifically, difficulties in focusing on a task may lead to the easier triggering of the behaviors associated with Facebook use or gaming under the influence of external and internal triggers. Consequently, this impulsivity dimension may have a significant influence on the mechanism of the development of problematic online behavior.

Additionally, among male gamers only, the present study’s findings may also indicate that thoughts unrelated to the task and intruding thoughts (cognitive instability) [63, 124]) may contribute to increased PVG. One possible explanation for this may be related to game transfer phenomena (GTP) which are defined as “involuntary phenomena manifesting as altered sensorial perceptions, automatic mental processes, actions and behaviors as a result of the transfer of experiences from the virtual to the real world” [125, p. 12]. GTP may (among other things) manifest as involuntary thoughts about videogames [126]. Consequently, it can be cautiously assumed that individuals with high cognitive instability may have more difficulty in refraining from thinking about the videogame despite doing other things. In this context, previous research [127] reported that GTP are associated with PVG. However, it should be noted that GTP are a broader issue related not only to the negative aspects of videogames playing but also to the positive aspects [128].

The present study also showed significant differences between male and female gamers in the relationship between PVG and cognitive instability (H7). One possible explanation for this difference may be related to the different meaning of videogames in the lives of males compared to females. More specifically, male gamers play videogames in person or online with friends more frequently than female gamers [129]. Additionally, male gamers look for different things in videogames than female gamers, which are often included in game designs, thereby enhancing male gamers’ abilities [130]. Additionally, female gamers face the sexual (mis)representation of female characters in the game, online harassment, and the expectation that they are less skillful players compared to male gamers [131]. Consequently, it can be assumed that videogames are more important for male gamers than female gamers, and males talk to each other about them more often. Consequently, taking into account the large amount of videogame-related content appearing in the environment (e.g., advertisements, conversations, etc.) and GTP [125, 126], male gamers with high cognitive instability may have more difficulty in restraining themselves from thinking about playing videogames, which eventually can be conducive to PVG. However, this assumption requires further investigation

The findings showed a positive relationship between perseverance (motor impulsivity subtype) and (i) PVG among female gamers, and (ii) PFU among male gamers. However, there was no statistically significant relationship between the motor (motor impulsivity subtype) and either PFU or PVG. Consequently, these results partially support H2. These results may indicate that instable lifestyle associated with perseverance impulsivity [63, 124] is probably connected with problematic online behaviors. One possible explanation for this difference may be related to coping strategy the stress of unstable life. More specifically, according to game motives [132], PVG is associated with coping among male gamers, and competition among female gamers. Additionally, escapism has been associated with PVG among both groups [133]. Analogically, Masur et al. [134] reported that PFU was associated with motives use such as escapism and socialization. Consequently, it can be assumed that individuals with high perseverance can easily treat gaming and Facebook use as a way of coping. This assumption can be supported by the negative relationship between perseverance and life satisfaction, irrespective of gender (see Figs 1 and 2). Another explanation may be related to the attention subtype of impulsivity. More specifically, attention and perseverance subtypes of impulsivity were associated with PVG among female gamers, whereas they were associated with PFU among male gamers. In this context, it can be assumed that difficulties in focusing on a task and unstable lifestyle may perhaps lead to frequent changes in individuals’ activity. More specifically, individuals may not be focused on one activity but look for and test out different activities.

Additionally, it can be assumed that frequent changes in activity can also contribute to lower life satisfaction. In this context, it should be noted that there was a gender difference in the relationship between perseverance and problematic online behaviors in the present study. There was a significant difference between male and female gamers in case of the relationship between the PFU and perseverance (H7). Also, there was a significant relationship between perseverance and PVG among female gamers. By contrast, this relationship was non-significant among male gamers. Nevertheless, there was no statistically significant difference between both groups in relation between perseverance and PVG utilizing the Wald’s test. Taking into account the association between attention and perseverance, female gamers may change activities and look for new ones, such as new videogames, whereas male gamers may look for non-game-related activities, such as using social media (e.g., Facebook). However, these explanations need to be verified in more detail, taking into account the motives for changes in gamers’ activity.

In the present study, the motor subtype of motor impulsivity, which is the tendency to acting quickly [63, 124] was not statistically associated with any problematic behaviors, irrespective of gender. Previous research [75] also reported no statistically significant relationship between motor impulsivity and PVG. Additionally, taking into account lack of premeditation which is the tendency to act without thinking and its association with motor impulsivity [25], previous research showed no statistically significant relationship between lack of premeditation and PFU [70] as well as PVG [75–77]. Consequently, it can be assumed that speedy decision-making is probably not associated with problematic online behaviors. However, taking into account other research indicating unclear associations between this impulsivity dimension and problematic online behaviors [78, 93, 97], further research should be carried out into this aspect of impulsivity in the context of problematic online behavior.

The results also showed that higher cognitive complexity (non-planning impulsivity subtype) was associated with PFU only among female gamers. Apart from this relationship, there was no other statistically significant relationship between the subtypes of non-planning impulsivity and both PFU and PGV among male and female gamers, which partially supported H3. Taking into account that non-planning impulsivity (the tendency think without deliberation [24, 63]), these results may be consistent with previous research [66, 67] which reported that PFU is not related with the reflective system which is connected with making deliberate judgments and evaluations and putting together strategic action plans for goal pursuit [68]. Additionally, previous research [17, 75, 79] showed no statistically significant relationship between non-planning impulsivity and problematic online behaviors. Consequently, it can be assumed that as a tendency to think without deliberation may be not be related to problematic behaviors such as PFU and PVG.

Additionally, the result of the present study showed a relationship between cognitive complexity and PFU among female gamers. This finding may indicate that enjoying mental challenges associated with cognitive complexity [63, 124] is probably associated with PFU among female gamers. One of the possible explanations may be related to boredom. More specifically, individuals with high cognitive complexity may have difficulty with boredom may treat Facebook use as a break from boredom. In this context, Orosz, Tóth-Király and Bőthe [135] postulated that one of the Facebook intensity dimensions is boredom. Additionally, Ryan et al. [136] noted boredom as one of the motives for using Facebook. Also, Phu and Gow [137] reported a positive association between the number of Facebook friends, daily time spent on Facebook, and boredom.

In this context, the explanation associated with boredom is also supported by the positive relationship between cognitive complexity and weekly time spent on Facebook (see Fig 1). Furthermore, the results of the present study showed a significant difference between male and female gamers in case of the relationship between PFU and cognitive complexity (H7). More specifically, this relationship was non-significant among male gamers. This result might be related to a higher level of Facebook intensity associated with boredom among females compared to males [137]. Consequently, it can be assumed that females with a higher level of cognitive complexity may have a greater intolerance to boredom than males, which may result in more frequent use of Facebook and higher PFU.

Additionally, findings showed a negative relationship between PVG and life satisfaction in both groups. However, PFU was positively associated with life satisfaction among male gamers. Consequently, these results partially support H4. In regard to PVG, these results are in line with previous research [17, 82–85]. Consequently, it can be assumed that problematic behavior associated with gaming may result in decreased life satisfaction. More specifically, gamers with a PVG may observe the losses associated with uncontrolled gaming (e.g., poor grades, loss of a job, deterioration of social relationships etc.). Consequently, they may feel that their lives are not the same as they want them to be.

Contrary to the hypothesis (H4), the findings showed a positive relationship between PFU and life satisfaction among male gamers. Previous studies have reported the opposite relationship (for review [19]). However, one study [87] reported that PVG negatively correlated with life satisfaction, whereas problematic social media use did not correlate with life satisfaction. Błachnio and Przepiorka [107] also reported no statistically significant relationship between PFU and life satisfaction. However, Błachnio et al. [138] reported a positive relationship between PFU and life satisfaction among Italians but not among Americans.

It should also be noted that Błachnio, Przepiorka and Pantic [112] presented a classification of three Facebook user groups: (i) ordinary Facebook users, (ii) intensive Facebook users, and (iii) addicted Facebook users. Ordinary Facebook users were characterized by high self-esteem and high life satisfaction, whereas intensive Facebook users were characterized by low self-esteem and high life satisfaction. However, addicted Facebook users were characterized by low self-esteem and low life satisfaction. Taking into account that females use Facebook more often than males [139] and females tend to have a higher level of PFU than males [140, 141], it can be assumed that males are more likely to be classified as intensive Facebook users [112]. However, this assumption requires further investigation.

The findings showed there was a relationship between impulsivity dimensions and life satisfaction, which confirms H5. More specifically, there was a negative relationship between cognitive instability, perseverance and life satisfaction, whereas the motor impulsivity subtype was positively associated with life satisfaction, irrespective of gender. In this context, the relationship between cognitive instability and life satisfaction was stronger among male gamers compared to female gamers. Additionally, there was a negative relationship between self-control, cognitive complexity, and life satisfaction only among male gamers. These results may indicate that thoughts unrelated to the task and intruding thoughts (cognitive instability) and instable lifestyle (perseverance) [63, 124] may contribute to decreased life satisfaction, irrespective of gender. Additionally, enjoying mental challenges (cognitive complexity) and lack of plans and thinking without deliberation (self-control) [63, 124] may lead to lower life satisfaction among male gamers.

These results are in line with previous research [27–30] indicating a negative relationship between impulsivity and life satisfaction. However, the results of the present study showed that speedy decision-making (motor) [63, 124] might be associated with increased life satisfaction among both groups. In this context, it should be noted that Arrindell, Heesink and Feij [36] showed a positive relationship between life satisfaction and impulsivity characterized as habitual response style of decision-making in ambiguous situations among males and females. Additionally, they found a negative relationship between life satisfaction and disinhibition characterized by the desire to find release through social disinhibition, drinking alcohol, and going to parties, irrespective of gender. Consequently, the results of the present study may in line with research by Arrindell, Heesink and Feij [36].

Burnett et al. [142] reported that individuals with high lack of premeditation performed better on the ‘Traffic Light’ task which requires participants to take risks under time pressure when trying to maximize rewards. Consequently, in some circumstances, a high lack of premeditation may be associated with positive performance and maximization of achievement. Taking into account that lack of premeditation is the tendency to act without thinking and previous research showed the association between lack of premeditation and motor impulsivity [25], it could be that individuals with high motor impulsivity achieve positive effects as a consequence of their decisions under specific circumstances, and consequently feel more satisfaction with life. However, more research is needed on this issue.

The findings showed no mediation effects between impulsivity dimensions and life satisfaction via PVG or PFU, and therefore did not confirm H6. Despite previous research examining the relationship between impulsivity and problematic behaviors [58, 64, 66, 67, 69], between impulsivity and life satisfaction [27, 28], and between problematic behaviors and life satisfaction [17–19], there were no mediation effects. These results may indicate that the indirect relationship is too weak to show statistically significant effects. Additionally, it can be assumed that there are two different mechanisms, one associated with impulsivity and life satisfaction and other associated with impulsivity and problematic behaviors. This assumption may be supported by the results of present study indicating that other dimensions of the impulsivity trait are related to life satisfaction rather than problematic behaviors.

Additionally, the findings showed a negative relationship between the attention subtype of impulsivity and the number hours spent gaming per week, whereas there was a positive relationship between cognitive complexity and the number hours spent gaming per week among male gamers. These findings may indicate that difficulty in focusing on current tasks and disliking mental challenges maybe contribute to lower time spent gaming. This assumption may be supported by research describing the structural characteristics of videogames [143–145] which may make demands on the gamers in order to achieve game results. Consequently, male gamers with low cognitive complexity and high attention subtype of impulsivity may be exploring new activities not related to the videogame.

The present study also showed that male gamers had a higher level of PVG and more time spent gaming per week compare to female gamers. This result is in line with previous research [20, 146]. Additionally, Hamlen [147] postulated that the increased gaming might lead to increased feelings of success and achievement, which then leads to increased playing time among male gamers. Also, male gamers have a stronger motivation for gaming than females, which has been associated with entertainment, seeking information, and social relationships [148]. In contrast, McLean and Griffiths [131] reported that female gamers played alone, played anonymously, and moved groups regularly because they often experienced a lack of support and harassment when gaming online alongside males. Taken together, male and female gamers may experience different preferences for the gratification they receive when gaming.

The results showed that two models out of eight were well matched to the data (see Table 3). One of the models assumed that time spent on Facebook and gaming were consequences of PFU and PVG, respectively, whereas the other assumed that PFU and PVG were consequences of time spent on Facebook and gaming hours, respectively. Taking into account information criteria, the model showing that time spent on Facebook and gaming were consequences of problematic behavior was more informative than the other model. In this context, some previous studies [97, 149] viewed time spent on Facebook or gaming as the predictors of problematic behavior. However, other research [98, 99, 150] viewed time spent on Facebook or gaming as the consequences of problematic behavior. According to I-PACE model, Brand et al. [15] postulated a shift from gratification to compensation during the addiction process. More specifically, in the beginning, individuals spend more and more time on Facebook or gaming because they may experience the gratification associated with the use of these media. However, when the gratification switches to compensation, the use of the medium will be more the result of problematic behavior. Consequently, this type of mechanism described in the I-PACE model could be the reason why two models had acceptable fits with the data (see Table 3). However, further research is needed to verify this assumption.

## Limitations and future research

The results presented in this study should be interpreted in light of several limitations. First, the purpose of the study was to examine the relationship between PFU, PVG, life satisfaction, and dimensions of impulsivity. Consequently, impulsivity was assessed by self-report methods rather than more objective behavioral and neuronal methods [151]. Therefore, the variables in the present study should be replicated utilizing non-self-report methods. Second, the study utilized a self-report method and was cross-sectional. Consequently, there are well-known methods biases when participants answer such questions (e.g., social desirability), and only longitudinal studies can provide insights into the causality of the significant associations found among the variables of interest. Third, the participants were all young Polish gamers, so the results are not representative of non-gamer groups and should be treated cautiously when referring to other cultures. Fourth, the internal validity of specific subscales of the BIS-11 (such as perseverance) were below the accepted norm in the present study. Consequently, it is important to be cautious regarding the interpretation of the results related to these subscales. Fifth, it should be noted that PFU is only one example of problematic SNS use [39]. Consequently, it is essential to be careful in generalizing the results to other SNS platforms, because Facebook users can play games, gamble online, watch videos, share photos, update their profiles, and message their friends [39, 40]. Finally, taking into account structural characteristics of videogames [143, 144], future studies should consider the relationship between impulsivity dimensions, videogames characteristics, and problematic behavior.

## Supporting information

S1 Fig Schemas of eight theoretical models. (PDF)

S1 Table The Kolmogorov-Smirnov test for normality. (PDF)

S2 Table Correlation coefficients between impulsivity dimensions. (PDF)
