# Supplementary figures and images for: Problematic Facebook use and problematic video gaming as mediators of relationship between impulsivity and life satisfaction among female and male gamers
Source: PLoS One. 2020 Aug 18;15(8):e0237610. doi: 10.1371/journal.pone.0237610 (PMC7437455; doi:10.1371/journal.pone.0237610)

# S1. Schemas of eight theoretical models:

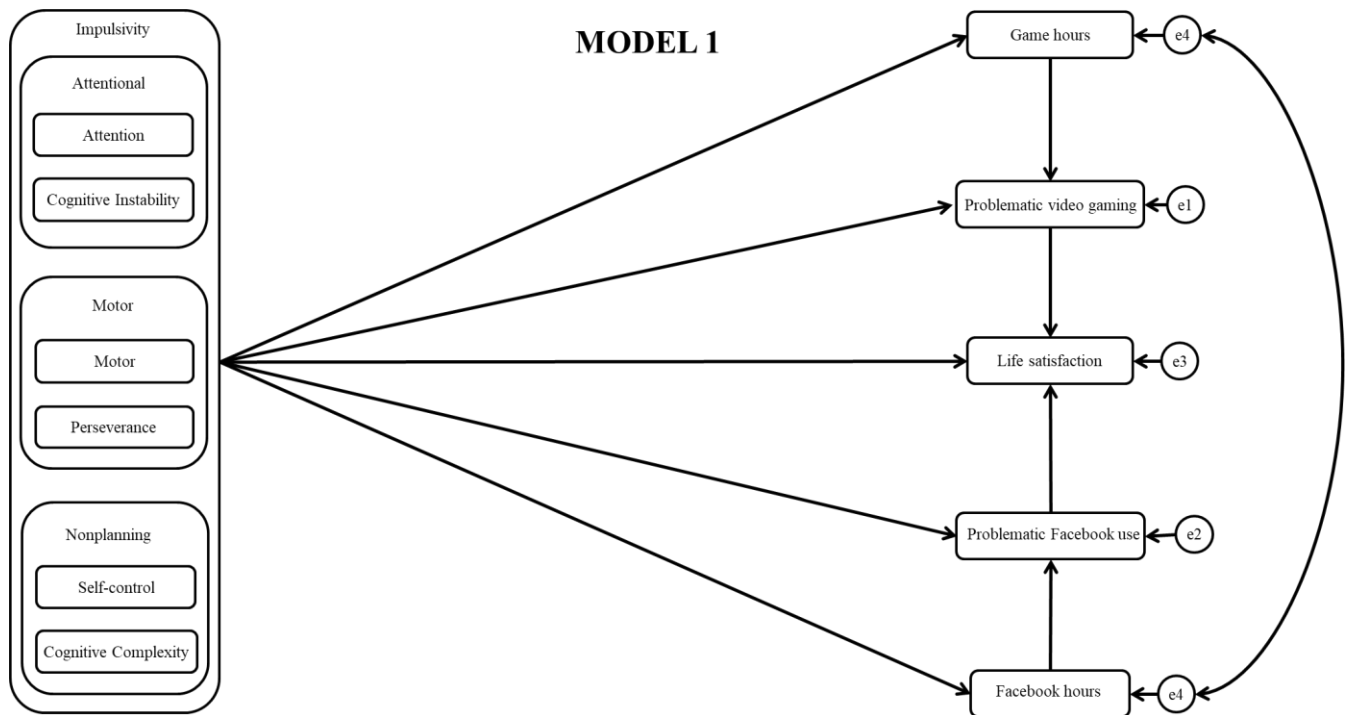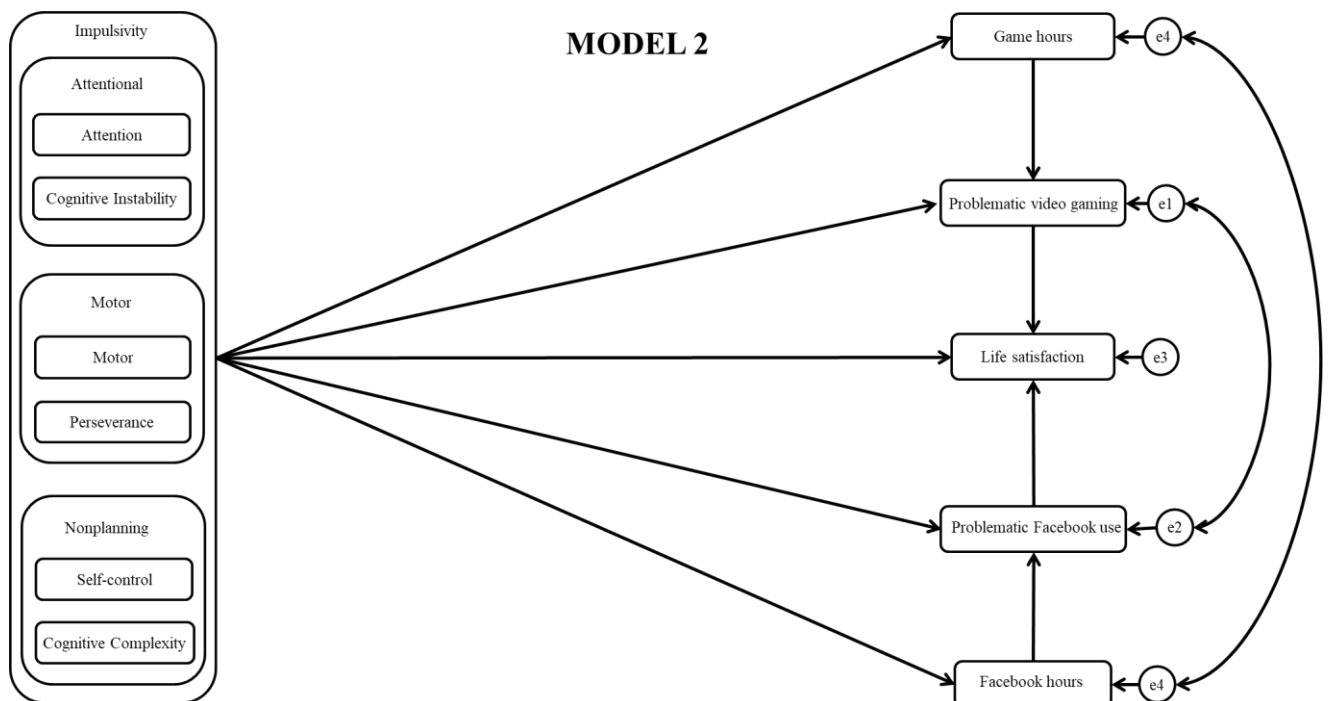

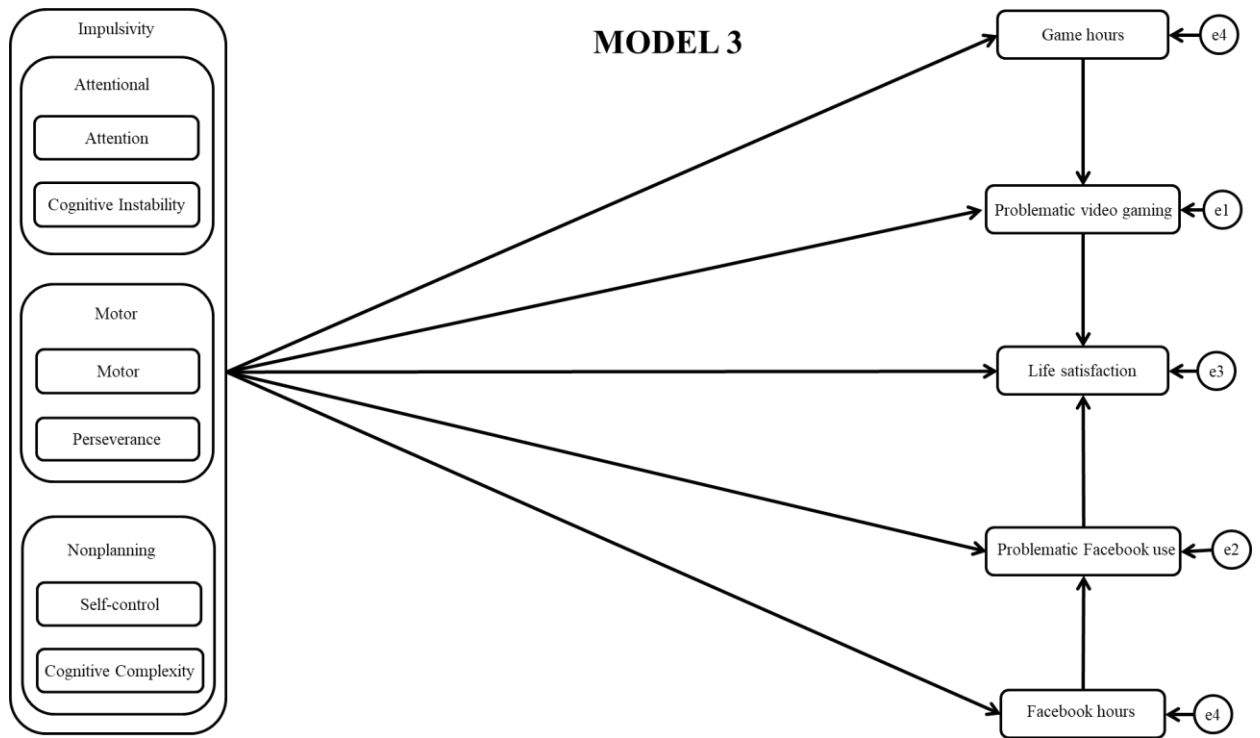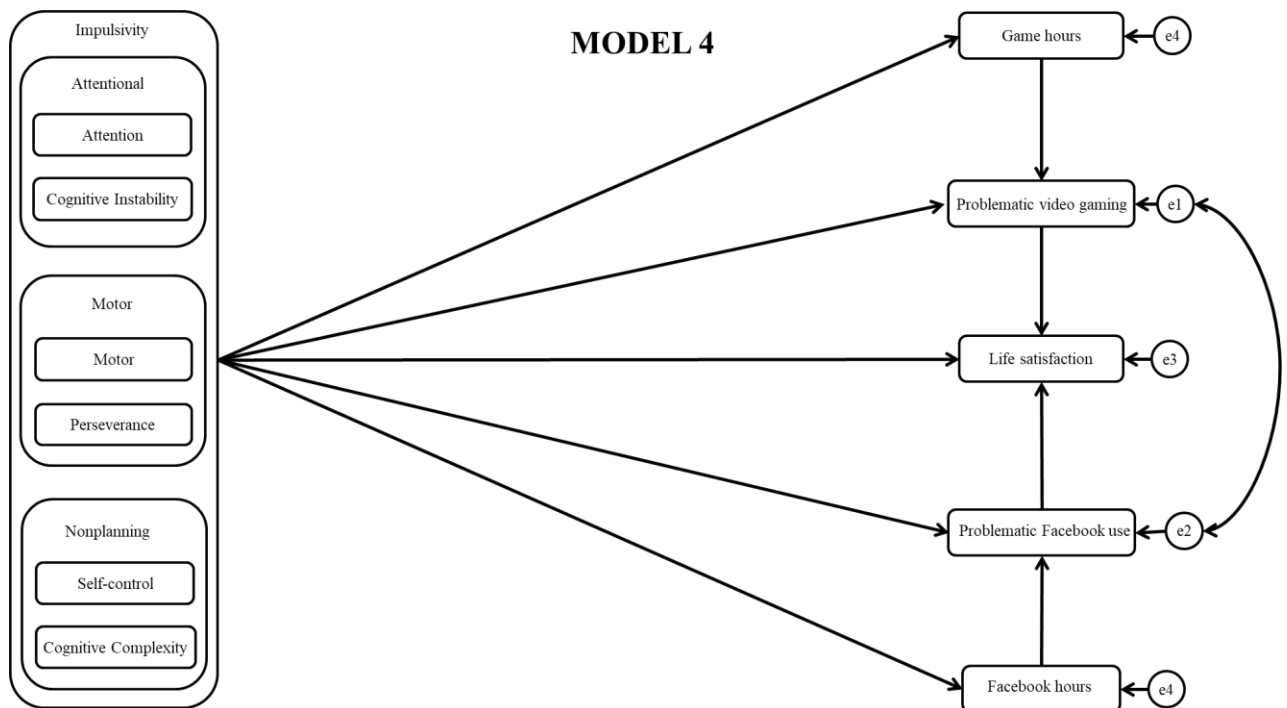

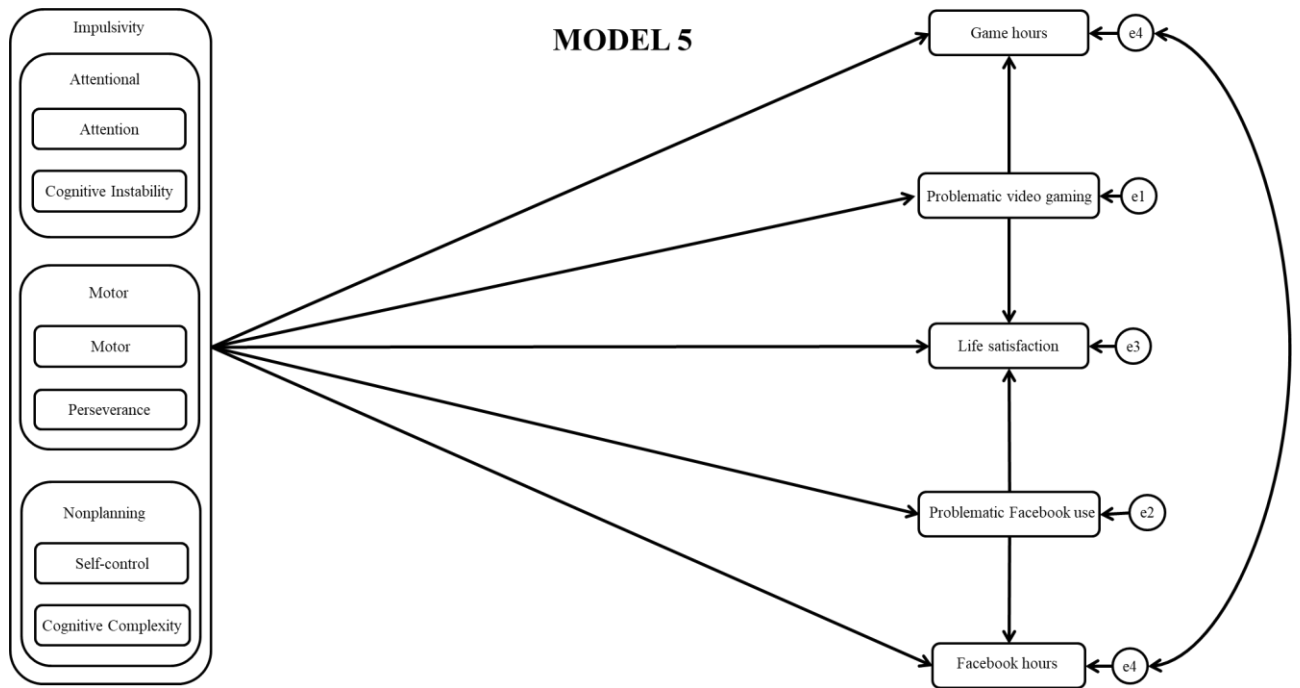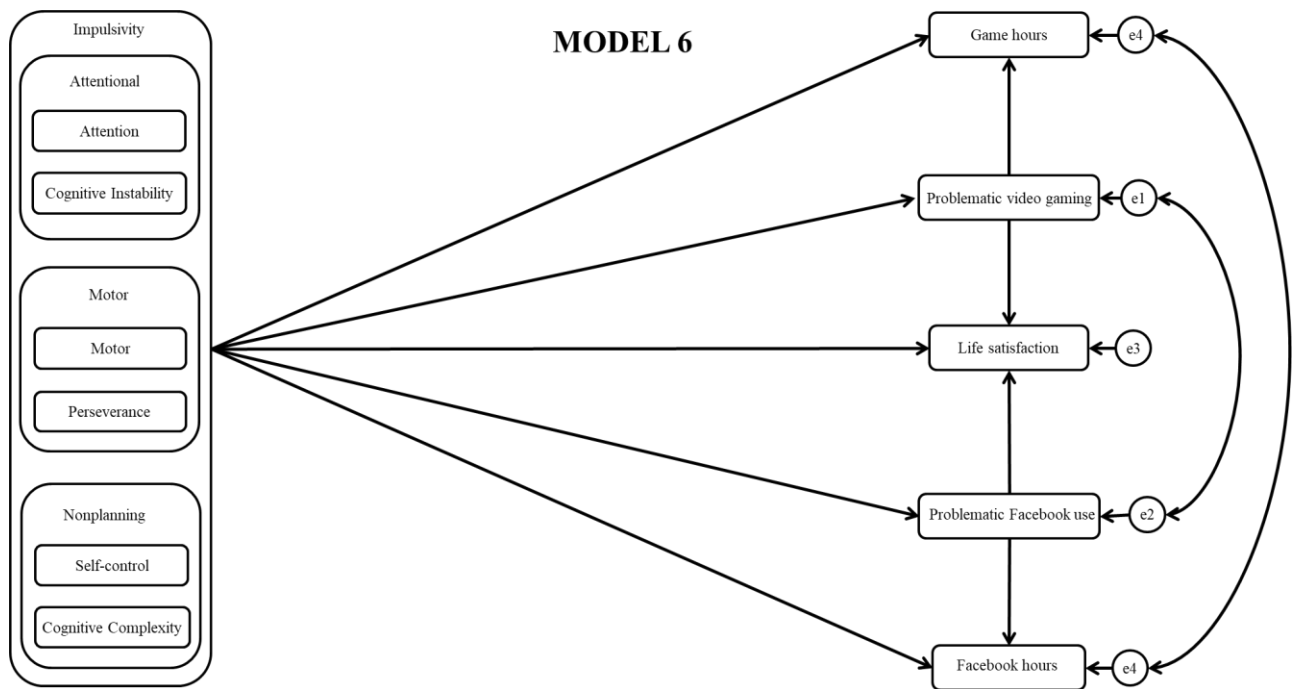

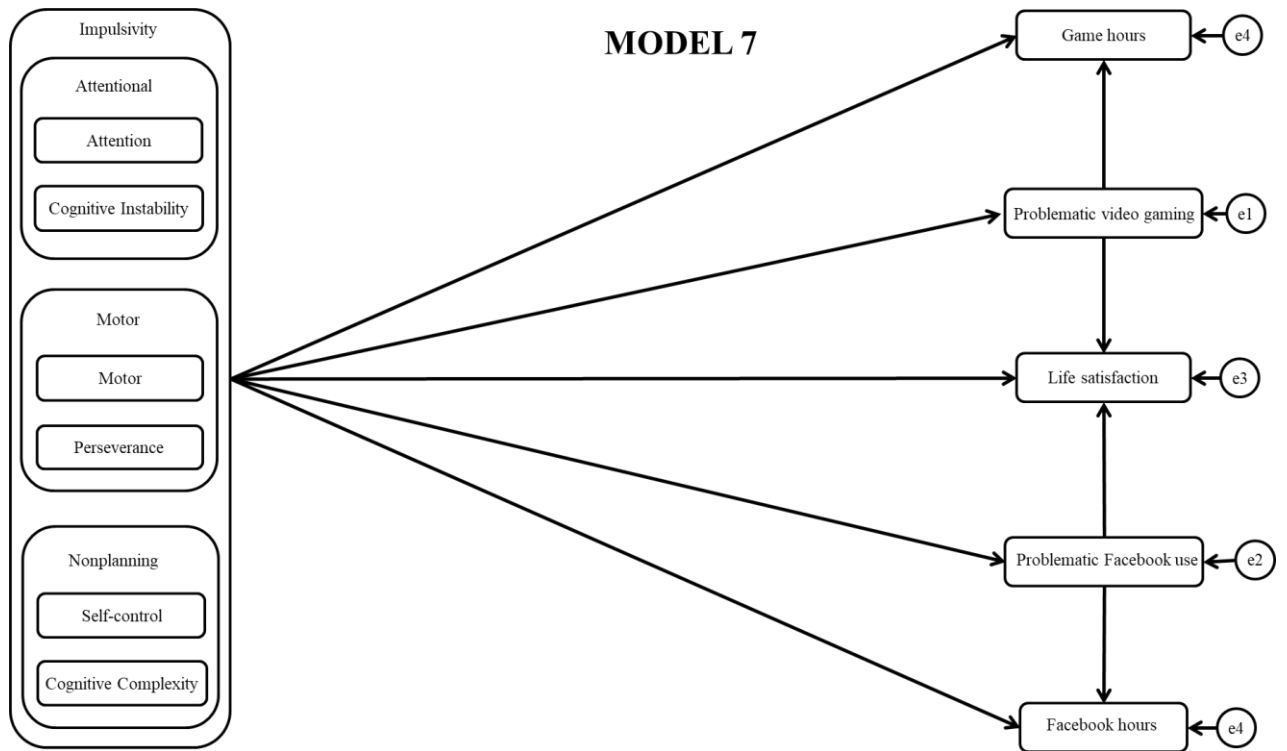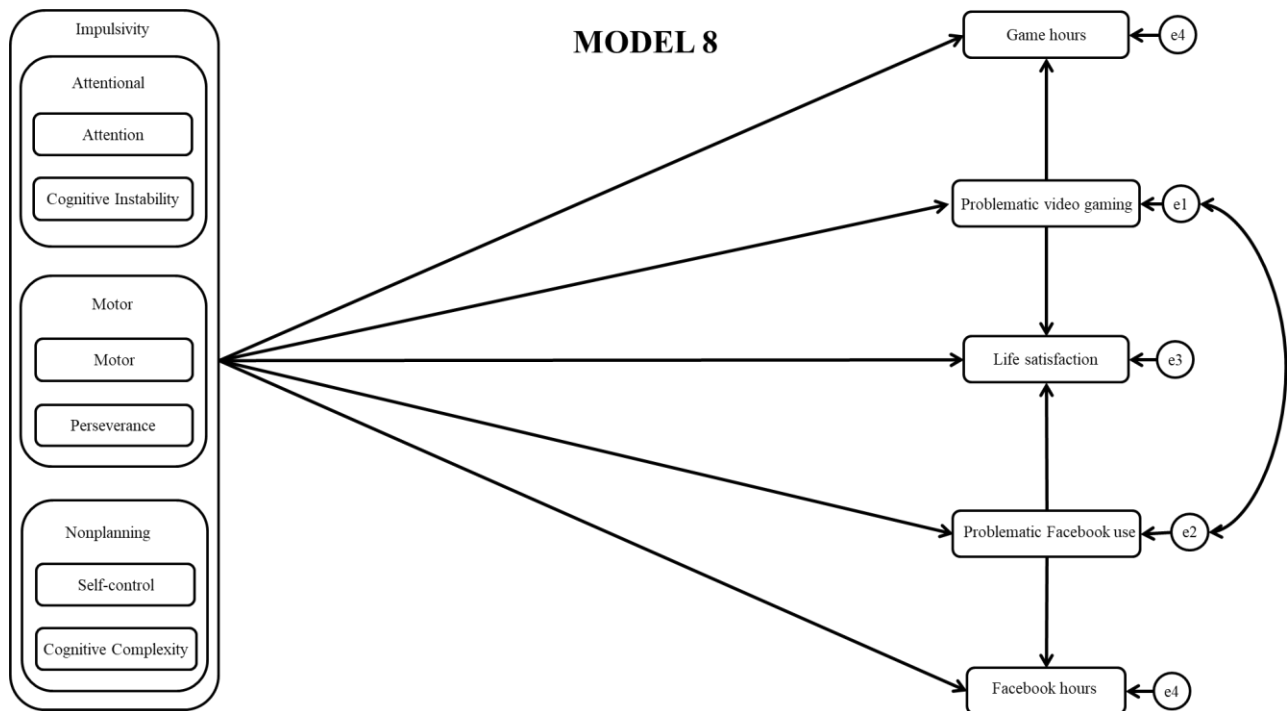

Supplement: S1 Fig — (PDF) [file pone.0237610.s001.pdf]
