# Supplementary material for: Problematic Facebook use and problematic video gaming as mediators of relationship between impulsivity and life satisfaction among female and male gamers
Source: PLoS One. 2020 Aug 18;15(8):e0237610. doi: 10.1371/journal.pone.0237610 (PMC7437455; doi:10.1371/journal.pone.0237610)
Supplement: S1 Table — (PDF) [file pone.0237610.s002.pdf]

S2. The Kolmogorov-Smirnov test for normality.

Variables		Male gamers (N = 282)		Female gamers (N = 391)	
		K-S	p<	K-S	p<
Problematic Facebook use		0.10	0.001	0.09	0.001
Facebook number of hours per week		0.23	0.001	0.26	0.001
Problematic video gaming		0.09	0.001	0.19	0.001
Game number of hours per week		0.18	0.001	0.29	0.001
Impulsivity	Attentional - Attention	0.14	0.001	0.11	0.001
	Attentional –Cognitive instability	0.13	0.001	0.12	0.001
	Motor - Motor	0.10	0.001	0.09	0.001
	Motor - Perseverance	0.17	0.001	0.17	0.001
	Nonplanning – Self control	0.10	0.001	0.09	0.001
	Nonplanning – Cognitive complexity	0.10	0.001	0.10	0.001
	Total	0.07	0.003	0.06	0,001
Life satisfaction		0.08	0.001	0.07	0.001
