# Supplementary material for: Problematic Facebook use and problematic video gaming as mediators of relationship between impulsivity and life satisfaction among female and male gamers
Source: PLoS One. 2020 Aug 18;15(8):e0237610. doi: 10.1371/journal.pone.0237610 (PMC7437455; doi:10.1371/journal.pone.0237610)
Supplement: S2 Table — (PDF) [file pone.0237610.s003.pdf]

S3. Correlation coefficients between impulsivity dimensions.

Variables/groups	r	SE	z	p	95%CI	
Attention, Cognitive instability						
Female gamers	0.24	0.05	5.11	0.001	0.15	0.33
Male gamers	0.39	0.05	8.34	0.001	0.30	0.48
Attention, Motor						
Female gamers	0.27	0.05	5.43	0.001	0.17	0.36
Male gamers	0.44	0.05	9.28	0.001	0.35	0.53
Attention, Perseverance						
Female gamers	0.29	0.04	6.41	0.001	0.20	0.38
Male gamers	0.40	0.05	7.65	0.001	0.30	0.50
Attention, Self-control						
Female gamers	0.38	0.05	8.03	0.001	0.29	0.47
Male gamers	0.49	0.05	10.57	0.001	0.40	0.58
Attention, Cognitive complexity						
Female gamers	0.31	0.04	6.92	0.001	0.22	0.39
Male gamers	0.45	0.05	9.01	0.001	0.35	0.55
Cognitive instability, Motor						
Female gamers	0.36	0.05	7.84	0.001	0.27	0.45
Male gamers	0.46	0.05	9.27	0.001	0.37	0.56
Cognitive instability, Perseverance						
Female gamers	0.25	0.05	5.44	0.001	0.16	0.34
Male gamers	0.17	0.06	3.00	0.003	0.06	0.28
Cognitive instability, Self-control						
Female gamers	0.14	0.05	2.90	0.004	0.05	0.24
Male gamers	0.25	0.05	4.78	0.001	0.15	0.35
Cognitive instability, Cognitive complexity						
Female gamers	0.02	0.05	0.40	0.690	-0.08	0.12
Male gamers	0.21	0.06	3.63	0.001	0.10	0.33
Motor, Perseverance						
Female gamers	0.27	0.05	5.75	0.001	0.18	0.37
Male gamers	0.42	0.05	7.82	0.001	0.32	0.53
Motor, Self-control						
Female gamers	0.55	0.04	14.58	0.001	0.47	0.62
Male gamers	0.49	0.05	9.13	0.001	0.38	0.59
Motor, Cognitive complexity						
Female gamers	0.24	0.05	4.86	0.001	0.14	0.34
Male gamers	0.42	0.05	8.37	0.001	0.32	0.52
Perseverance, Self-control						
Female gamers	0.23	0.05	4.40	0.001	0.13	0.33
Male gamers	0.32	0.05	5.89	0.001	0.21	0.42
Perseverance, Cognitive complexity						
Female gamers	0.30	0.04	6.92	0.001	0.22	0.39
Male gamers	0.39	0.05	7.31	0.001	0.29	0.50
Self-control, Cognitive complexity						
Female gamers	0.29	0.05	6.04	0.001	0.20	0.38
Male gamers	0.46	0.04	10.33	0.001	0.37	0.55
